# Dosimetric Comparison Study Between Free Breathing and Breath Hold Techniques in Patients Treated by Liver-Directed Stereotactic Body Radiation Therapy

**DOI:** 10.7759/cureus.40382

**Published:** 2023-06-13

**Authors:** Atsuto Katano, Tomoyuki Noyama, Kosuke Morishima, Yuki Nozawa, Hideomi Yamashita

**Affiliations:** 1 Radiology, The University of Tokyo Hospital, Tokyo, JPN

**Keywords:** dosimetric comparison study, breathing technique, liver metastases, radiotherapy, free breathing, liver-directed sbrt, liver, dose comparison, hepatocellular carcinoma, sbrt

## Abstract

Background

Breathing motion management is the key to delivering stereotactic body radiation therapy (SBRT) for liver lesions. This study aimed to compare the dosimetric parameters of liver SBRT using two different techniques: free breathing and breath hold.

Method

The study included 11 patients with liver metastases or hepatocellular carcinoma who underwent liver-directed SBRT. A dosimetric comparison was performed using dose-volume histogram analysis, evaluating parameters such as the maximum dose to 5 cc of bowel volume, mean liver dose (MLD), and liver V20 and V30. Statistical analyses were performed to compare results.

Results

The findings revealed that the breath hold technique resulted in significantly lower doses to the bowel and smaller volumes of normal liver tissue receiving 20 Gy (V20) and 30 Gy (V30) than the free breathing. Although there was no statistically significant difference in the MLD between the two techniques, the breath hold technique resulted in a lower MLD.

Conclusion

This dosimetric comparison study suggests that the breath hold technique is associated with lower radiation exposure to the bowel and normal liver tissues. Although this may not be feasible for all patients, it may be an appropriate procedure for selected individuals. Further research is needed to validate these findings in different patient populations and explore their impact on clinical outcomes and patient-reported quality of life.

## Introduction

Stereotactic body radiation therapy (SBRT) is a radiation therapy technique that delivers a high dose of radiation to a tumor in a few fractions while minimizing the dose to the surrounding normal tissue. For early-stage non-small-cell lung cancer (NSCLC), Chang et al. recently reported the long-term results of the revised STARS trial, which compared SBRT with surgery in 80 patients with NSCLC treated using a dose of 54 Gy in three fractions or 50 Gy in four fractions [1]. The primary endpoint was the three-year overall survival rate, and non-inferiority could be claimed if the three-year overall survival rate after SBRT was lower than that after surgery by ≤12%. This study found that long-term survival after SABR was non-inferior to that after video-assisted thoracoscopic surgical lobectomy with mediastinal lymph node dissection for operable early-stage NSCLC. SBRT is an established alternative to surgery for early-stage lung cancer.

Liver-directed SBRT is a well-established treatment option for patients with primary or secondary liver tumors, with excellent local control rates reported in several studies [2]. The precision of liver SBRT is largely attributed to its ability to target tumors accurately, which is made possible by using imaging techniques to track the movement of the target during the respiratory cycle [3].

Breathing motion is a major source of uncertainty during liver SBRT, as the liver moves with respiration, and this movement can also cause the tumor to move [4]. To address this issue, various techniques have been developed to manage the breathing motions of the liver during treatment. Free breathing and breath hold are two commonly used techniques for liver SBRT [5]. Although both have been used in clinical practice, their dosimetric differences in liver SBRT have yet to be thoroughly investigated [6].

Therefore, this study aimed to compare the dosimetric parameters of liver SBRT using free breathing and breath hold technique. Dosimetric parameters to evaluate the dose to critical structures. The results of this study will help improve clinical decision-making regarding the choice of breathing technique for liver SBRT.

## Materials and methods

This retrospective study included consecutive patients with liver metastases or hepatocellular carcinoma (HCC) who underwent liver-directed SBRT at the University of Tokyo Hospital, Tokyo, Japan, between March 2020 and May 2021. This study was approved by the Research Ethics Committee of the Faculty of Medicine of the University of Tokyo (Approval number: 3372(6)), and the need for informed consent was waived owing to the retrospective nature of the study. The inclusion criteria were as follows: age >18 years, Eastern Cooperative Oncology Group (ECOG) performance status of 0-1, no prior radiation therapy to the liver, and the availability of treatment planning data for both free breathing and the breath hold technique. Patients with respiratory motion amplitudes of >1 cm were excluded.

All patients were immobilized in the supine position with both arms up and underwent four-dimensional computed tomography (4D-CT) and breath hold CT simulations. The 4D-CT images were sorted into 10 phases based on the respiratory phase signal, and the internal target volume (ITV) was defined as the union of the gross tumor volume (GTV) contoured on each phase. The planning target volume (PTV) was generated by adding a 5 mm isotropic margin to the ITV. The prescribed dose covered at least 50% of PTV volume. Pinnacle treatment planning systems have been used for radiotherapy planning.

Two treatment plans were generated for each patient: free breathing and breath hold technique. The free breathing plan was generated using the average CT of the 10-phase 4D-CT, whereas the breath hold plan was generated using end-expiratory CT or end-inspiratory acquisition during breath-holding. For the breath hold technique, the patients were instructed to inhale deeply and hold their breath for 15-20 seconds during the acquisition of breath-holding CT.

A dosimetric comparison of organs at risk was performed using dose-volume histogram analysis. The bowel included duodenum, jejunum, and ileum. Normal liver volume was calculated as PTV subtracted from whole liver volume. The following dose-volume parameters were evaluated for both plans: maximum dose to 5 cc (D5cc) of bowel volume, mean liver dose (MLD), and liver V20 and V30. Liver V20 and V30 were defined as the percentages of the liver volume receiving at least 20 and 30 Gy, respectively.

Statistical comparisons were performed to estimate treatment characteristics. The Wilcoxon signed-rank test was used to compare continuous variables between the free breathing and breath hold technique plans. The significance level was set at p <0.05. All statistical analyses were performed using the R software (R Foundation for Statistical Computing, Vienna, Austria).

## Results

Eleven patients were enrolled in the dosimetric comparison study. The patient characteristics are summarized in Table 1.

**Table 1:** Patient characteristics: clinical features and diagnostics of patients assessed in this study ECOG: Eastern Cooperative Oncology Group; PS: performance status; HCC: hepatocellular carcinoma

ID	Age	Sex	Disease	Location	Tumor diameter (mm)	ECOG-PS	Child-Pugh	Dose and fractionation
A	76	Male	primary HCC	S8	22	0	5	48Gy/4fr
B	83	Male	primary HCC	S8	42	0	5	50Gy/10fr
C	53	Female	metastatic lesion	S3	44	0	5	50Gy/10fr
D	77	Female	primary HCC	S8	18	0	7	48Gy/4fr
E	85	Female	metastatic lesion	Hilum lymph node	29	1	5	36Gy/12fr
F	59	Male	primary HCC	IVC	27	0	6	50Gy/10fr
G	70	Male	primary HCC	S8	25	1	6	48Gy/4fr
H	73	Male	primary HCC	S4/8	24	0	5	48Gy/4fr
I	70	Male	primary HCC	S7	25	0	5	48Gy/4fr
J	73	Male	primary HCC	S6	18	0	5	48Gy/4fr
K	67	Male	primary HCC	S1	16	0	5	60Gy/10fr

The median age of the study population was 73 years (range 53-85 years), and the mean tumor diameter was 29 cm (range 16-44 cm). The median ECOG-performance status (PS) was 0 (range 0-1). Most of the target lesions were primary HCC (81.8%). Based on the Child-Pugh classification, 10 (90.9%) patients were classified as A and 1 (9.1%) as B. The mean prescribed dose was 48 Gy (range 36-60 Gy) in 4-10 fractions.

**Figure 1:**
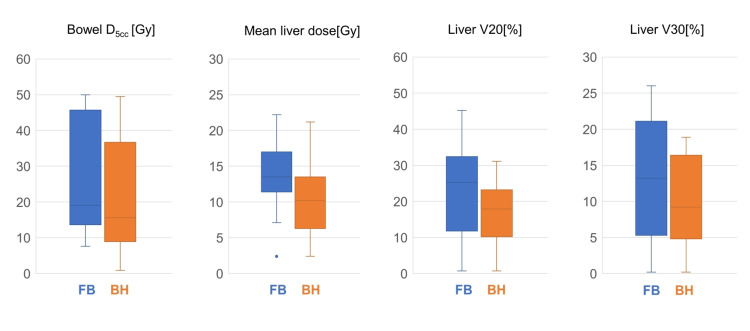
Boxplot of the dosimetric parameters estimated for free breathing and breath hold technique . FB: free breathing; BH: breath hold technique

The dosimetric parameters of free breathing and the breath hold technique are shown in Figure 1.

The breath hold technique resulted in a significantly lower median dose to bowel D5cc than free breathing (15.6Gy; SD: 16.8 vs 19.6Gy; SD: 16.2, respectively) (p <0.001). The breath hold technique was associated with a significantly lower normal liver tissue volume receiving 20 Gy (V20) and 30 Gy (V30) than free breathing (9.2 %; SD: 5.9 vs 13.2%; SD: 8.3, respectively) (p =0.006). Although there was no statistically significant difference in the MLD between the two groups, the breath hold technique resulted in a lower MLD than free breathing (p =0.053).

## Discussion

The results of this study indicate that the breath hold technique is associated with lower radiation exposure in the bowel and normal liver tissue. These findings are consistent with previous studies demonstrating the benefits of using the breath hold technique for liver-directed SBRT. Thaper et al. retrospectively analyzed 33 cases of liver SBRT for HCC and concluded that there was a significant difference in the radiation dose received by the normal liver between free breathing and the breath hold technique [7]. The breath hold technique reduces respiratory motion, resulting in the sparing of normal tissue.

In free breathing technique, the patient breathes normally during treatment, and radiation is delivered based on the respiratory motion [8]. In contrast, the breath hold technique involves instructing patients to hold their breath at a specific point in the respiratory cycle, typically during the exhalation phase while radiation is delivered [9]. Dosimetric comparisons between these techniques are important to determine which technique is more effective in minimizing the dose to the surrounding normal tissues to reduce radiation-induced adverse events. Future studies should be needed to compare our results to other motion management modalities such as gating and tracking methods in liver SBRT.

However, it should be noted that the breath hold technique may not be feasible for all patients, particularly those with poor pulmonary function or difficulty in holding their breath [10]. In addition, the breath hold technique may be associated with longer treatment times due to the need for multiple breath holds during treatment delivery [11]. This may be challenging for some patients, particularly those who experience discomfort or anxiety during the procedure. Hardcastle et al. insisted that the choice of technique should consider both motion management effectiveness and patient comfort, as these significantly affect treatment time [12].

Another potential limitation of this study is the use of a single treatment planning system and delivery technique. The dosimetric differences observed in this study may not be generalizable to other treatment planning systems or delivery techniques. And, the PTV target coverage of 50% was low compared to previous studies. Further studies are needed to confirm these findings and investigate the feasibility and efficacy of the breath-holding technique in different patient populations.

## Conclusions

This dosimetric comparison study demonstrated that the breath hold technique is associated with lower radiation exposure to the bowel and normal liver tissues compared to free breathing. Although the breath hold technique may not be feasible for all patients, particularly those with poor pulmonary function or difficulty holding their breath, it may be an appropriate option for selected patients. Further studies are needed to confirm these findings and investigate the feasibility and efficacy of the breath hold technique in different patient populations. Moreover, the impact of these dosimetric differences on clinical outcomes and patient-reported quality of life should be confirmed in the future.
